# The Relationship Between Health-Related Social Needs and Screening Mammography Among Women Seeking Care at a Federally Qualified Community Health Center Network

**DOI:** 10.1089/whr.2024.0059

**Published:** 2024-09-26

**Authors:** Carla Salazar, Lacey Johnson, Paula Carcamo, Paula Rusca, Bridget G. Magner, Josephine Llaneza, Natalie Rodriguez, Andrew Cooper, Mita Sanghavi Goel

**Affiliations:** ^1^ Division of General Internal Medicine, Northwestern University Feinberg School of Medicine, Chicago, Illinois, USA.; ^2^ Erie Family Health Centers, Chicago, Illinois, USA.

**Keywords:** health-related social needs, screening mammography, federally qualified, community health center, women

## Abstract

**Purpose:**

In this study, we examined the relationship between health-related social needs (HRSNs) and screening mammography.

**Methods:**

We gathered data from April 2020 to February 2021 among women ages 52–74 years at a federally qualified community health center network in the Chicago region. We measured HRSNs using a one-item screener, and among those screening positive, with an eight-item questionnaire. Screening mammography was measured as (1) ever having mammography and (2) mammography completed in the past 2 years. We examined the relationship between HRSNs in the one-item and multi-item questionnaires and both measures of screening mammography using logistic regression.

**Results:**

Among 3711 women, mean age was 60 years, 68% were Hispanic/Latino, 62% were best served in Spanish, 39% had no insurance, and 71% had incomes <100% federal poverty level. In total, 32% reported an HRSN in the one-item screener. Of these, 74% completed the multi-item questionnaire; changes in income (60%) and inadequate access to food (46%) were the most common HRSNs reported. Overall, 65% reported prior mammography and 47% reported mammography in the past 2 years. There was an association between prior mammography and the one-item screener (odds ratio = 0.83, 95% confidence intervals = 0.70, 0.98), but no association between recent mammography and HRSNs reported in the one-item, specific HRSNs, or number of HRSNs.

**Conclusions:**

We found an association between ever having a mammogram and a positive one-item screener, but not in relation to specific HRSNs. The findings of this study may inform future assessments of HRSNs and understanding their relationships with preventive health care.

## Introduction

Numerous federal and state initiatives, such as those spearheaded by the Centers for Medicare and Medicaid Services (CMS)^1^ and the National Academy of Science, Engineering, and Medicine (NASEM)^2,3^ have focused on addressing social determinants of health (SDOH) and health-related social needs (HRSNs) within health care settings. SDOH, defined by the World Health Organization as “the non-medical factors that influence health oucomes,” encompasses the conditions in which people are born, grow, work, live, and age.^4^ Conversely, HRSNs are defined by CMS as “individual-level adverse social conditions that can negatively impact a person’s health or health care,” including food insecurity, housing instability, unemployment, safety needs, financial strain, social isolation, stress, lack of education, and lack of access to transportation.^5^

These factors are widely acknowledged to exert a significant influence on health and often serve as barriers to accessing and receiving health care services, including essential screenings such as mammography.^6–8^ For example, one study found that health care cost and/or no insurance was the most commonly reported barrier among women undergoing screening mammography.^9^ Another found housing concerns and lacking a regular provider predicted poor mammography uptake.^10^ Furthermore, one study found that mammography use decreased with an increasing number of reported HRSNs, not having a personal health care provider, lost or reduced hours of employment, receiving food stamps, lacking reliable transportation, feeling socially isolated, and cost.^11^ One strategy addressing these barriers was the development of patient navigation, which successfully improved breast cancer survival among a predominantly Black population in Harlem.^12–16^ This approach provided free and reduced-cost breast exams and an individualized one-on-one relationship that assisted individuals to access mammograms and follow-up services. While the operationalization of navigation has been broad in scope and could incorporate addressing health system barriers, educational barriers, and psychosocial barriers, one specific hypothesis has been that addressing HRSNs may have contributed to its success.

Yet, despite such interventions, the precise impact of HRSNs on screening mammography remains unclear, particularly within safety net clinical settings. Therefore, in this study, we aimed to determine the prevalence of HRSNs in the adult patient population eligible for screening mammography at Erie Family Health Centers (Erie) and examine the relationship of HRSNs with the use of screening mammography.

## Methods

### Study population

We conducted this study at Erie, which is a network of federally qualified community health centers with 13 sites across Chicago and nearby suburban regions. Erie serves more than 82,000 patients annually, including 71% Hispanic/Latino, 62% female, 47% best served in Spanish, and 90% living in low-income households. Approval for this study was obtained from the Northwestern University Institutional Review Board Office.

### Study inclusion/exclusion criteria

To accurately examine a population for whom screening mammography should have been completed at least once and for whom ongoing screening mammography would be recommended, our inclusion criteria for the study were: (1) women 52–74 years of age, (2) English or Spanish-speaking, (3) seeking care from Internal Medicine, Family Medicine, or Obstetrics/Gynecology at any Erie location, and (4) completing a telemedicine visit between April 2020 and February 2021. Exclusion criteria were personal history of breast cancer and not having a one-item screener at a telehealth visit.

### Measures

#### Predictor variables

Sociodemographic characteristics were reported by patients and recorded in the electronic health record (EHR). These included race (American Indian/Alaska Native [including American Indians or Alaska Natives of Latino/Hispanic descent], Asian, Black or African American [including Black or African American of Latino/Hispanic descent], Native Hawaiian, Other Pacific Islander, White [including Whites of Latino/Hispanic descent]), ethnicity (Hispanic or Latino, not Hispanic or Latino, Other or Undetermined), gender (Female, Male, Trans Female, Trans Male, Other), age, and preferred language (English, Spanish, Other).

We categorized race and ethnicity into one variable to prevent overlap and to combine categories with too few members to allow for analysis: Hispanic/Latino, White non-Hispanic/Latino, Black or African American non-Hispanic/Latino, and Other non-Hispanic/Latino. Given our inability to identify males, trans individuals, or others who might require mammography screening, we selected only individuals who self-identified as females in the gender variable.

Data on insurance type were reported by the insurance plan name. We categorized insurance into the following categories: uninsured, Medicaid, Medicare, Medicare–Medicaid, and private. Similarly, federal poverty level (FPL) was reported in granular numbers, and we created categories corresponding to the sliding scale payment plan at Erie (<100% FPL, 101–200% FPL, >200% FPL).

#### Outcomes measures

The primary outcome measure for this study was the completion of screening mammography. We measured this in two ways: ever screening mammography and up-to-date screening mammography. Ever screening mammography (Yes/No) was determined by whether mammography had ever been completed prior to the initial one-item screener. Up-to-date screening mammography (Yes/No) was determined by whether mammography was completed in the 2 years preceding the initial one-item screener.

### HRSNs screening variables

To develop HRSNs screening tool, we convened key stakeholders from Erie, including clinicians and staff, as well as academic partners with expertise in HRSNs screenings. This team reviewed existing HRSN screeners and identified questions for pilot testing among a sample of patients and staff. After obtaining feedback, we finalized an eight-item questionnaire to be used for in-person visits among a study population of 200 patients. Prior to the study launch, the COVID-19 pandemic disrupted clinical operations, and clinical care transitioned to a fully virtual platform. Furthermore, Erie implemented HRSN screening for all patients, not only the original study population. As a result of these changes, Erie streamlined processes to expedite the screening of all patients and created a one-item screener (Would you like a member of our care team to reach out to better understand your needs and connect you with support?), which was asked following a preface about the new initiative to identify nonclinical barriers to care and used for all patients seen for telehealth visits. All patients who answered “Yes” to the one-item screener received a follow-up call from Erie staff to administer an eight-item questionnaire composed of the following questions with “Yes,” “No,” or “Choose not to respond” as answer choices:

1. Has there been a change in your or your family’s income due to COVID-19?
2. Are you having trouble paying your rent right now?
3. Are you worried about having a safe and reliable place to sleep every night?
4. Do you have trouble taking care of a child?
5. Are you unable to get food when you are hungry?
6. Are you unable to get medications that you need?
7. Do you want resources to help you cope with stress?
8. Are there any needs you have that we have not discussed?

For this study, the multi-item questionnaire was categorized into the following eight HRSN domains: income, rent, safe place to sleep, childcare, access to food, access to medications, stress, and other.

#### HSRNs screening workflow

As a part of Erie’s telehealth previsit workflow, the one-item screener was asked by telehealth support assistants or providers before or at the beginning of a visit. Those responding “Yes” to the one-item screener were contacted over the phone within 1–120 days by a staff member who administered the eight-item HRSN questionnaire. The staff member then provided patients with requested resources based on responses to screening questions. The eight-item questionnaire required 5–7 minutes, and discussions to share resources took 15–20 additional minutes. Two phone calls and one text outreach were attempted for all participants screening positive on the one-item screener before they were marked as unsuccessful. Results for the one-item screener, multi-item questionnaire, and referral to resources were entered into the electronic medical record.

### Statistical analysis

In addition to descriptive analyses of the study sample, we examined the proportion of individuals responding “Yes” to the one-item screener. Among those responding “Yes” to the one-item screener, we also examined responses to the multi-item questionnaire. For the latter, we examined individual responses to each of the questions by domain (i.e., income, rent, safe place to sleep, childcare, access to food, access to medications, stress, and other), as well as the total number of affirmative responses to the multi-item questionnaire.

#### One-item screener analysis

To examine the relationship between HRSNs identified in the one-item screener and mammography completion, we performed unadjusted and adjusted for race/ethnicity logistic regression using ever and up-to-date mammography as outcome measures. The referent group for all logistic regression analyses was those answering “No” in the one-item screener. The 95% confidence intervals (CI) outside of the null value (odds ratio [OR] = 1) were considered statistically significantly different.

#### Multi-item questionnaire analysis

##### HRSNs domains

To investigate the association between individual HRSNs identified in the multi-item questionnaire and mammography, we performed unadjusted and adjusted for race/ethnicity logistic regression models using ever and up-to-date mammography as outcome measures. To ensure comparisons differentiated those with HRSNs from those without, we excluded participants who responded “Yes” to the one-item screener but responded “No” to all questions on the multi-item questionnaire, and we used those who answered “No” in the one-item screener as the referent group.

##### Number of affirmative responses

To examine the relationship between the total number of affirmative responses to the multi-item HRSNs questionnaire and mammography completion, we performed unadjusted and adjusted for race/ethnicity linear regression models using ever and up-to-date mammography as outcome measures. In these models, HRSNs were treated as continuous variables, and we limited them to participants who completed both the one-item and multi-item screeners. The referent group was those who answered “No” in the one-item screener.

## Results

### Demographics

We examined data from 3711 women ages 52–74 years; mean age was 60 years, most were Hispanic/Latino, best served in Spanish, had public forms of insurance, and had incomes <100% of FPL (Table 1).

**Table 1.** Demographics of Women 52–74 Years Old at Erie

	Women 52–74 (*n* = 3711)	
Age (years)		
Mean (SD)	60.2	(6)
	*N*	%
Race/ethnicity		
Hispanic/Latino	2531	68
White non-Hispanic/Latino	472	13
Black or African American non-Hispanic/Latino	347	9
Other non-Hispanic/Latino	284	8
Unknown	77	2
Language		
English	1321	36
Spanish	2,290	62
Other	100	2
Insurance type		
Uninsured	1443	39
Medicaid	1377	37
Medicare	482	13
Medicare–Medicaid	99	3
Private	309	8
Missing	1	0
Federal poverty level		
<100%	2644	71
101–200%	816	22
>200%	226	6
Missing	25	1

Erie, Erie Family Health Centers; SD, standard deviation.

### Health-related social needs

#### One-item screener

Most eligible individuals were administered the one-item screener (*n* = 3711), of whom 32% (*n* = 1181) screened positive for having an HRSN, 65% (*n* = 2415) screened negative for having an HRSN, and 3% (*n* = 115) did not respond.

#### Multi-item questionnaire

Of those that reported an HRSN in the one-item screener (*n* = 1181), 74% (*n* = 874) completed the multi-item questionnaire, of whom 82% reported at least one HRSN (Table 2). Specifically, 29% answered “Yes” to one question in the multi-item questionnaire, 28% answered “Yes” to two questions, and 17% answered “Yes” to three questions. The most commonly reported HRSNs were income (60%) and access to food (46%) (Table 2).

**Table 2.** HRSNs Reported on Multi-Item Questionnaire Among Patients Who Answered Yes at One-Item Screener

	*N*	%
Multi-item questionnaire administered		
Yes	874	100
Number of Qs answered “Yes”		
0	153	18
1	249	29
2	248	28
3	147	17
4	50	6
5	18	2
6	6	0
7	3	0
Income (Q1)		
Yes	522	60
No	305	35
Missing	47	5
Rent (Q2)		
Yes	141	16
No	680	78
Missing	53	6
Safe place to sleep (Q3)		
Yes	130	15
No	687	79
Missing	57	6
Childcare (Q4)		
Yes	18	2
No	794	91
Missing	62	7
Access to food (Q5)		
Yes	399	46
No	428	49
Missing	47	5
Access to medications (Q6)		
Yes	119	14
No	697	80
Missing	58	6
Stress (Q7)		%
Yes	21	2
No	158	18
Missing	695	80
Other needs (Q8)		
Yes	183	21
No	636	73
Missing	55	6

HRSNs, health-related social needs.

### Mammography rates

Of the 3711 women ages 52–74 years old, 65% (*n* = 2408) had at least one mammogram prior to the initial HRSN screening and 47% (*n* = 1755) had up-to-date mammography.

### Logistic and linear regression analyses

#### One-item screener

We found a statistically significant association between HRSNs identified in the one-item screener and ever screening mammography in the unadjusted model (OR = 0.83, 95% CI = 0.70, 0.98), and adjusted for race/ethnicity model (OR = 0.74, 95% CI = 0.61, 0.88).

However, we did not find a significant association between HRSNs in the one-item screener and up-to-date screening mammography in the unadjusted model (OR = 1.04, 95% CI = 0.90, 1.19) and adjusted for race/ethnicity model (OR = 0.95, 95% CI = 0.82, 1.10).

#### Multi-item questionnaire

##### HRSN domains

We did not find a statistically significant association between individual HRSNs reported in the multi-item questionnaire and ever mammography or up-to-date mammography in the unadjusted and adjusted for race/ethnicity models (Tables 3 and 4).

**Table 3.** Association Between Up-to-Date and Ever Screening Mammography and Individual HRSNs on Multi-Item Questionnaire (Unadjusted)

	Multi-item questionnaire domains	Unadjusted OR (95% CI)	
		Ever mammography	Up-to-date mammography
One-item screener No^a^			
	Income	1.03 (0.81, 1.32)	0.84 (0.70, 1.02)
	Rent	0.80 (0.53, 1.22)	0.81 (0.58, 1.14)
	Safe place to sleep	0.95 (0.57, 1.56)	0.86 (0.60, 1.22)
	Childcare	1.48 (0.45, 4.86)	0.81 (0.33, 2.00)
	Access to food	0.99 (0.75, 1.31)	0.83 (0.68, 1.03)
	Access to medications	0.97 (0.58, 1.63)	0.81 (0.57, 1.17)
	Stress	0.67 (0.26, 1.70)	0.69 (0.30, 1.58)
	Other needs	1.28 (0.88, 1.85)	1.11 (0.83, 1.50)

^a^ Referent group.

CI, confidence intervals; OR, odds ratio.

**Table 4.** Association Between Up-to-Date and Ever Screening Mammography and Individual HRSNs on Multi-Item Questionnaire Adjusted for Race/Ethnicity

	Multi-item questionnaire domains		^b^Adjusted OR (95% CI)	
		Ever mammography	Up-to-date mammography	
One-item screener No^a^			
	Income	1.19 (0.92, 1.53)	0.95 (0.79, 1.16)
	Rent	0.92 (0.60, 1.42)	0.90 (0.63, 1.26)
	Safe place to sleep	1.05 (0.62, 1.78)	0.94 (0.65, 1.34)
	Childcare	1.28 (0.36, 4.50)	0.82 (0.32, 2.07)
	Access to food	1.16 (0.87, 1.54)	0.94 (0.76, 1.17)
	Access to medications	1.13 (0.66, 1.93)	0.92 (0.63, 1.33)
	Stress	0.77 (0.29, 2.00)	0.76 (0.33, 1.76)
	Other needs	1.29 (0.88, 1.89)	1.16 (0.86, 1.58)

^a^ Referent group.

^b^ Adjusted for race/ethnicity.

CI, confidence intervals; OR, odds ratio.

##### Number of affirmative responses

We did not find a statistically significant association between the number of questions answered “Yes” in multi-item questionnaire and ever screening mammography. There were no statistically significant results in the unadjusted model (OR = 1.11, 95% CI = 0.98, 1.26) nor the adjusted race/ethnicity model (OR = 1.09, 95% CI = 0.95, 1.23). Similarly, there was no association with up-to-date screening mammography, in the unadjusted model (OR = 1.0, 95% CI = 0.91, 1.09) and adjusted for race/ethnicity model (OR = 0.98, 95% CI = 0.89, 1.07).

## Discussion

This study found that women who reported HRSNs in the one-item screener are less likely to have ever had a mammogram compared to women who did not report HRSNs, even after adjusting for race/ethnicity. There was, however, no association between HRSNs and recent mammography. Furthermore, we found no association between specific HRSNs, or number of HRSNs, and any measure of mammography.

This study adds to a body of literature examining the relationship between HRSNs and associated interventions on breast health. Starting in the early 1990s, Freeman and colleagues developed and studied a community-based intervention that combined patient navigation through financial and social barriers with access to free and reduced-cost screening mammography.^13–16^ They found this intervention significantly increased mammography screening and decreased late-stage cancer detection.^12^ Subsequent studies of patient navigation programs that addressed social factors, including The Boston Racial Ethnic Approaches to Community Health,^10^ among others,^17,18^ found that addressing social, logistic, and other barriers led to increases in uptake of mammography. These studies, however, have not examined clinic-based HRSN screening as has been endorsed by the National Academies of Medicine.^19^ More recent studies examining social drivers of health have found a variety of relationships emerging between addressing HRSNs and outcomes, including depression, emergency department utilization, and immunization rates, though they did not examine relationships with mammography.^20–22^

Our study extends the literature in several important ways. First, we focused on a largely understudied population in an unstudied urban setting—a largely Hispanic/Latino patient population in the Chicago region. As health risks and their relationships to health outcomes may vary by geography and race/ethnicity, providing data regarding these risks across populations is critical. We did not find that housing concerns, as measured by the ability to afford rent and having a safe place to sleep, were related to mammography use, as others have.^10^ Similarly, though our study population was larger than in either of the prior studies, we did not find any relationship between any specific HRSNs and mammography use. As resources required to identify and address HRSNs can be substantial, determining the most effective and efficient methods for doing so is crucial for sustaining these practices.^23^ It is possible that the acuity and impact of housing concerns and other HRSNs vary by region and that approaches to these barriers should be regionalized and not generalized.

Furthermore, our findings deepen the understanding of the relationship between HRSNs and health outcomes. A recent evaluation of the Accountable Health Communities model, which examined the effects of social risk screening on emergency room use, found that screening for HRSNs decreased emergency room visits.^24^ Surprisingly, however, the model did not decrease the prevalence of HRSNs themselves. These findings indicate that the mechanism by which the social care provided in clinical settings may influence health outcomes may be more complex than simply via the reduction of HRSNs. Furthermore, it is unclear whether specific needs, such as transportation or housing, had the strongest association with health outcomes, which in this study was emergency department utilization. Our study supports the finding that the presence of any HRSN—not a specific HRSN or number of HRSNs—is important to assess and is most strongly associated with lack of care, namely prior mammography. As health systems implement methods of assessing HRSNs, this finding highlights the potential importance of a global assessment of social needs, in addition to the assessment of specific domains.

Our study findings also strike a contrast to findings from a 2020 study of nearly 400,000 women presenting for mammography across three geographic regions.^9^ That study found that women who had experienced a prior lapse in screening were more likely to report experiencing barriers such as competing responsibilities, transportation, cost of care, or coverage of care.^9^ Our study differs from this prior study population in that ours was conducted among patients seen at health centers, not among those presenting for mammography services. Also, in contrast to other studies, the rates of screening in our population were lower than the most recent studies, with only 47% completing mammography within the preceding 2 years. These findings demonstrate the importance of examining HRSNs within primary care settings and not solely at the point of mammography screening.

## Limitations

Our study has several limitations. First, there is no standardized method for assessing HRSNs, making comparisons across studies challenging. It is possible that our one-item measure and specific measures for housing concerns, food insecurity, and stress captured different HRSNs than those in other studies. Second, our data on HRSNs were gathered during the initial year of the COVID-19 pandemic. This could have influenced the nature and acuity of HRSNs gathered in a manner that was not representative of HRSNs preceding the pandemic or at present, though ongoing data collection indicates these HRSNs persist. It also led to delays in being able to reach patients screening positive on the one-item screener, with an average time of 20 days between the one- and multi-item questionnaires. During that time, risks may have changed and expanded social services may have been available. The COVID-19 pandemic also led to a decrease in mammography, which may have influenced our ability to identify a relationship between HRSNs and recent mammography; however, in our time trend analyses, we did not find statistically significant differences between patients with and without HRSNs at specific quarters, similar to our full cohort. Third, none of the health centers included in this study have on-site mammography services and may have incomplete capture of mammography. All patients cared for at Erie are referred to mammography care at locations outside of the Erie system. As a result, mammography is conducted and recorded primarily at outside entities and may not be accurately captured within the Erie EHR system. The Erie data used in this study, however, is the same source of data used for quality metrics to clinicians and population health managers, as well as federal reporting purposes. Last, this iteration of HRSNs screening did not capture transportation barriers, as at the time of this study, a large portion of clinical operations were conducted via telehealth services, and transportation barriers were not perceived as critical to address at the time. As mammography services were exclusively delivered at external sites, however, it is possible that we did not capture an influential barrier.

Despite these limitations, this study had numerous strengths, including its large Hispanic/Latino population and substantial capture, ∼51%, of HRSNs data among all patients seeking telehealth care. Our findings emphasize the importance of capturing local data on HRSNs as well as global assessments of HRSNs to allow for the provision of support that is not adequately captured in our limited assessments of specific HRSNs.

## Conclusion

As evidence of the impact of HRSNs on potential disparities in health behaviors and outcomes continues to mount, it becomes increasingly imperative to identify the prevalence of various HRSNs among adult patients, as well as to explore their association with the utilization of screening mammography. Our study shows that women indicating HRSNs in a single-item screener were less likely to have ever undergone mammography compared to those who did not report HRSNs, even after adjusting for race/ethnicity. Future investigations should aim to differentiate between HRSNs captured in a one-item versus a multi-item screener and assess whether clinic-based interventions could enhance the receipt of recommended preventive care.

## Abbreviations Used

CMS: Centers for Medicare and Medicaid Services
EHR: electronic health record
Erie: Erie Family Health Centers
FPL: federal poverty level
HRSNs: health-related social needs
NASEM: National Academy of Science, Engineering, and Medicine
SDOH: social determinants of health
